# Supplementary material for: Tau Internalization is Regulated by 6-O Sulfation on Heparan Sulfate Proteoglycans (HSPGs)
Source: Sci Rep. 2018 Apr 23;8:6382. doi: 10.1038/s41598-018-24904-z (PMC5913225; doi:10.1038/s41598-018-24904-z)
Supplement: Supplementary file 1 — Supplementary Data [file 41598_2018_24904_MOESM1_ESM.docx]

**Tau Internalization is Regulated by 6-O Sulfation on Heparan Sulfate Proteoglycans (HSPGs)**

Jennifer N. Rauch^1^, John J. Chen^2^, Alexander W. Sorum^3^, Gregory M. Miller^3^, Tal Sharf^1^, Stephanie K. See^2^, Linda C. Hsieh-Wilson^3^, Martin Kampmann^2^, Kenneth S. Kosik^1,*^

^1^Neuroscience Research Institute, Department of Molecular Cellular Developmental Biology, University of California, Santa Barbara, CA 93106, USA.

^2^Institute for Neurodegenerative Diseases, Department of Biochemistry & Biophysics, The California Institute for Quantitative Biomedical Research, Quantitative Biosciences Institute, University of California, San Francisco, and Chan Zuckerberg Biohub, San Francisco, CA 94158, USA.

^3^Division of Chemistry and Chemical Engineering, California Institute of Technology, Pasadena, CA 91125

*Correspondence: [kenneth.kosik@lifesci.ucsb.edu](mailto:kenneth.kosik@lifesci.ucsb.edu)

**Supplemental Figures and Legends**

**Supplemental Figure 1.** (**a**) Uptake of 2N4R monomer is a time regulated process in H4 cells. (**b**) Uptake of 2N4R quaternary structures in ReN-VM neural progenitor cell line (1 h at 37°C). Identical control experiments were performed at 4°C and subtracted from 37°C data to generate the curves shown. (**c**) Uptake of 2N4R quaternary structures in SH-SY5Y neuroblastoma cells (1 h at 37°C). Control experiments performed at 4°C were subtracted to generate the curves shown. For uptake experiments, three independent experiments were performed in duplicate with a representative experiment shown and the error shown is SEM. (**d**) Immunocytochemistry of H4 cells after 2N4R monomer uptake (1 h at 37°C or 4°C). Green represents 2N4R-488, while total tau protein is shown in red. DAPI was used to stain nuclei. (**e**) Immunocytochemistry of iPS-derived neurons at day 14 of maturation, showing that they are positive for the neuronal markers MAP2 and tau. DAPI was used to stain nuclei.

**Supplemental Figure 2.** (**a**) qPCR of sgRNA knockdown of selected hit genes. First bar represents non-targeting sgRNA negative control, followed by gene specific sgRNA. Some genes had two sgRNAs chosen, in which case both are shown. Results are the average of three independent experiments performed in triplicate, and error bars represent SD. (**b**) Uptake of 2N4R monomers (50 nM) in H4i cells with selected gene knockdowns, normalized to a WT (no sgRNA) control. (**c**) List of genes that decreased or increased uptake of tau and their respective effect on the cell cycle. (**d**) TP53 gene knockdown shortens the cell cycle and increases cell growth. (**e**) PD033, a small molecule that stalls cells in G1 phase, increases uptake of tau. (**f**) Uptake of 2N4R monomers (50 nM) in H4i or DNM2 knockdown cells +/- treatment with Dynasore (50 μM) inhibitor. All uptake experiments were performed in duplicate over three independent experiments with the data combined. Lines on the graphs represent WT mean +/- 3 standard deviations and error bars represent SEM. NT sgRNA stands for non-targeting sgRNA.

**Supplemental Figure 3.** (**a**) Excitatory post synaptic potentials (EPSPs) of mouse brain slice cultures. (**b**) Internalization of 2N4R-488 into mouse slice culture in the presence of various heparin derivatives. Hoechst stain is used to label nuclei. (**c**) Quantification of the median Hoechst fluorescence intensity for tau uptake in mouse slice culture. Two independent experiments were performed with multiple images (>5) from each condition. Error bars represent SEM. (**d**) qPCR of Sulf1 and Sulf2 overexpression. Results are the average of three independent experiments performed in triplicate, and error bars represent SD. (**e**) Heparan sulfate from H4 cells in which Sulf1 or Sulf2 is overexpressed as compared to WT transfected cells was purified, digested and AMAC-labeled as described in methods. Fold-change of overall disaccharides found in Sulf1 and Sulf2 overexpression lines relative to WT. (**f)** Fold-change of disaccharides bearing 6-*O*-sulfate modifications found in Sulf1 and Sulf2 overexpression lines relative to WT. Experiments were performed in triplicate, error bars represent SD, and a two-way ANOVA analysis with Dunnett’s method was used to determine significance compared to WT control (*=p-value ≤0.05, **=p-value ≤0.01, ***=p-value ≤0.001, ns = not significant).
